# Dachengqi decoction for the treatment of acute pancreatitis: a comprehensive analysis based on metabolites, pharmacokinetics, and metabolites efficacy mechanisms

**DOI:** 10.3389/fphar.2025.1549909

**Published:** 2025-05-30

**Authors:** Wen Guo, Xiaobin Zhang, Xin Zhou, Wangpeng Lan, Jianqin Liu, Yali Liu, Li Li, Zhi Li

**Affiliations:** ^1^ Department of Spleen and Stomach Diseases, The Affiliated Traditional Chinese Medicine Hospital, Southwest Medical University, Luzhou, China; ^2^ The Key Laboratory of Integrated Traditional Chinese and Western Medicine for Prevention and Treatment of Digestive System Diseases of Luzhou city, The Affiliated Traditional Medicine Hospital, Southwest Medical University, Luzhou, China; ^3^ Research Center of Integrated Chinese and Western Medicine, The Affiliated Traditional Chinese Medicine Hospital, Southwest Medical University, Luzhou, China; ^4^ School of Integrated Traditional Chinese and Western Clinical Medicine, North Sichuan Medical College, Nanchong, China

**Keywords:** DaChengQi decoction, acute pancreatitis, traditional Chinese medicine, metabolite, efficacy mechanisms

## Abstract

Dachengqi decoction (DCQD) is a traditional Chinese medicine formula consisting of three botanical drugs and one mineral drug. It has anti-inflammatory, gastrointestinal motility, and microcirculation improvement effects. A large number of studies have shown that DCQD has a significant impact on the treatment of acute pancreatitis (AP). However, owing to the multi-component and multi-target characteristics of traditional Chinese medicine, it is difficult for modern scientists to understand the role of DCQD in treating AP. Therefore, We sorted out the literature data related to DCQD published in databases such as Web of Science, PubMed, and CNKI, and summarized the main metabolites of DCQD, including anthraquinones, flavonoids, lignans and other metabolites. The pharmacokinetic characteristics of the main metabolites entering the blood circulation are preliminarily summarized. Combining chemical analysis and network analysis, some metabolites in DCQD, such as emodin, rhein, and luteolin, may play important roles in the treatment of AP, and this review also summarizes the efficacy mechanisms of these metabolites. In brief, we have summarized the treatment of AP with DCQD from the aspects of metabolites, preliminary pharmacokinetic analysis, and efficacy mechanisms, to facilitate further understanding of the material basis of DCQD.

## Graphical Abstract

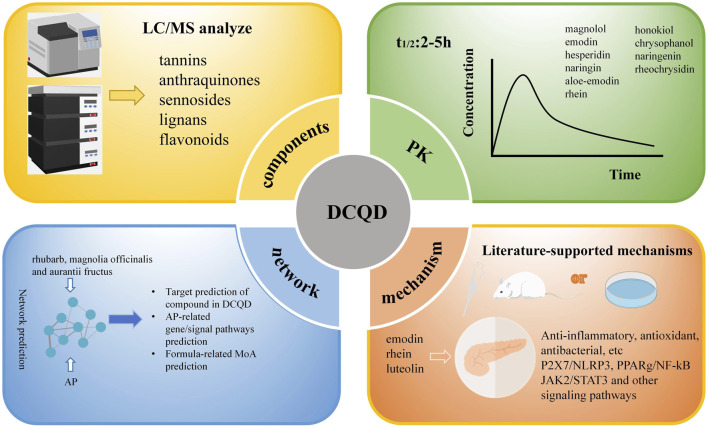

## Introduction

Traditional Chinese medicine (TCM) has multi-component and multi-target effects, and multiple components usually produce therapeutic effects through synergistic or antagonistic interactions (Song et al., 2013). DCQD is a classic traditional Chinese medicine prescription. It was first recorded in ‘*Shang-Han-Lun*’ in the Eastern Han Dynasty. It consists of four natural drugs *Rheum officinale Baill [*Polygonaceae*, Rhei Radix et Rhizoma](dahuang)*, *Magnolia officinalis Rehder and E.H.Wilson [*Magnoliaceae*,Magnoliae Officinalis Cortex](houpo), Citrus aurantium L[*Rutaceae*, Fructus Aurantii Immaturus](zhishi)* and *Natrii Sulfas(mangxiao)*. The four drugs are composed by a mass ratio of 4:8:3:1. DCQD belongs to a family of purgative herbal formulas widely used in China for the treatment of AP (Ma et al., 2020). However, due to the complex composition of traditional Chinese medicine prescriptions, the material basis of DCQD in the treatment of acute pancreatitis has not been fully clarified.

Elucidating the chemical entity of DCQD can facilitate elucidating its therapeutic principle for AP. With the development of analytical technology, high-performance liquid chromatography-mass spectrometry (LC-MS) has significant advantages in the analysis of TCM because of its advantages of accurate mass, high resolution, and high sensitivity, and it has been widely used in qualitative and quantitative studies of complex metabolites of TCM (Tang et al., 2022). Combined with this technology, DCQD has been detected to contain various metabolites, such as aloe-emodin, rhein, emodin, chrysophanol, magnolol, hesperidin, naringenin and naringin. Pharmacokinetics is a discipline that studies the absorption, distribution, metabolism and excretion (ADME) of drugs. The proportion of TCM metabolites entering the blood is low, therefore, performing pharmacokinetic studies is difficult. Combined with LC-MS technology, pharmacokinetic studies of dozens of metabolites in DCQD can be carried out simultaneously. Network analysis integrates the action networks of TCM and its targets, conducts network analysis on the drugs in DCQD and AP diseases, and predicts the metabolites, targets, and related pathways of DCQD in the treatment of AP.

Previous studies on the efficacy of DCQD in treating AP have shown promising results. However, these studies often lack a comprehensive analysis of the pharmacokinetics and pharmacodynamics of the active metabolites. Additionally, the synergistic or antagonistic interactions between different components of DCQD remain poorly understood. This review aims to address the gaps by providing a detailed analysis of the metabolites, pharmacokinetics, and efficacy mechanisms of DCQD. To ensure a comprehensive review, we employed a systematic search strategy using the keywords ‘Dachengqi Decoction’ and ‘acute pancreatitis’ in databases such as Web of Science, PubMed, and CNKI. Articles published from 2008 to 2023 were included in our analysis. The selection of studies was based on predefined inclusion and exclusion criteria to ensure the relevance and quality of the data.

### Metabolites in DCQD

**FIGURE 1:**
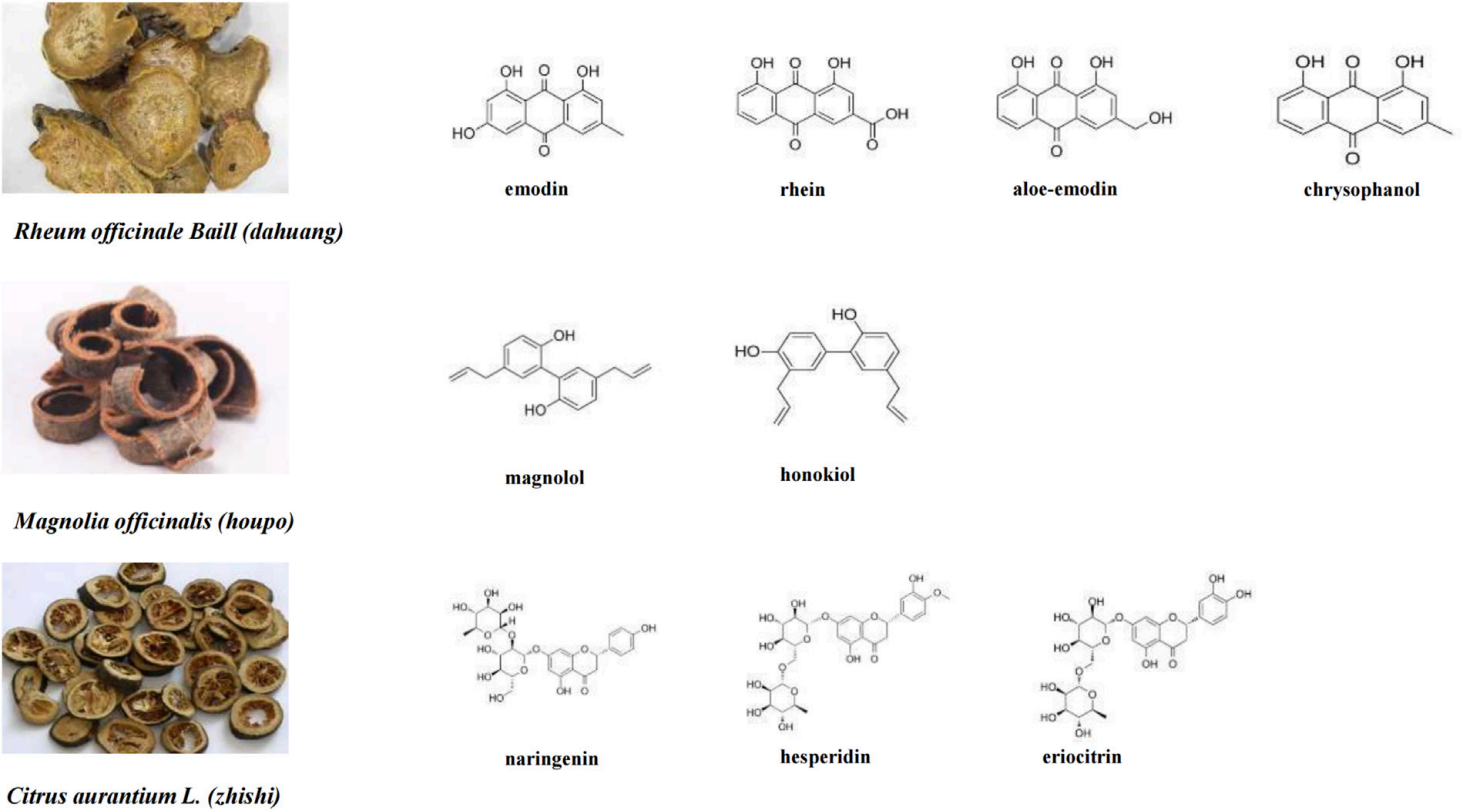
Chemical compositions of DCQD and the structures of the main compounds.

Elucidating the chemical composition of traditional Chinese medicine prescriptions and their interactions is the key to their development (Wolfender et al., 2015). Figure 1 shows the composition of DCQD. *Rheum officinale Baill,* which is derived from the dried roots and rhizomes of *Rheum palmatum L.* and *Rheum tanguticum Maxim.,* belongs to the genus *Rheum* in the family Polygonaceae. It contains anthraquinone metabolites such as emodin, aloe-emodin, chrysophanol, rheochrysidin and rhein (Zhuang et al., 2020). Studies have shown that rhubarb extract has antibacterial, gastrointestinal function regulatory, anti-inflammatory, and laxative effects, and can be used to treat acute pancreatitis (Xiang et al., 2020).

*Magnolia officinalis Rehder and E.H.Wilson,* a member of the Magnoliaceae family*,* is a traditional botanical drug derived from the dried bark, root bark, and branch bark of *M. officinalis* var. *Biloba.* It mainly contains phenolic metabolites, such as magnolol and honokiol (Poivre and Duez, 2017). It has anti-inflammatory, intestinal mucosal protective, and gastrointestinal dysfunction-relieving effects (Song et al., 2023), and can be used to treat digestive system diseases.

*Citrus aurantium L.,* a member of the Rutaceae family*,* is a traditional botanical drug derived from the dried immature fruit of the *sour orange* and its cultivated varieties. The main metabolites are flavonoids such as eriocitrin, naringenin, naringin, hesperidin, and limonin (Bai et al., 2018). It has anticancer, antioxidant, antiviral, vascular protective, and anti-inflammatory activities (Li et al., 2016).

*Natrii Sulfas* is a sulfate mineral used in traditional Chinese medicine. Its main compound is sodium sulfate decahydrate, followed by small amounts of sodium chloride, magnesium sulfate, calcium sulfate, and other inorganic salts. The main compounds of *Natrii Sulfas* are not absorbed in the intestine and cannot be measured in the blood. They mainly regulate the absorption of other metabolites and are not indicated in the TCM database (Nong et al., 2019). The pharmacological effects of *Natrii Sulfas* in the body are mainly anti-inflammatory and detumescent, promoting gastrointestinal motility, and regulating the intestinal flora (Tao et al., 2023).

However, the metabolites of TCM are complex, and chemical reactions may occur during the decoction process, which may change the chemical composition of the TCM metabolites (Wang et al., 2017). Therefore, the chemical composition of DCQD cannot be equated with the simple addition of those traditional Chinese botanical drugs.

In one study, DCQD was prepared, and 1 mL of liquid was mixed with 1 mL of DMSO. After extraction, centrifugation, and filtration, the chemical composition of DCQD was studied via the LC-ESI/MS/MS method. In negative ion mode, 37 metabolites were detected, and 14 metabolites were identified by comparison with standards. Among all the metabolites, 20 were derived from *dahuang*, 11 from *zhishi*, and six from *houpo*. These metabolites include tannins, anthraquinones, sennosides, lignans, and flavonoids (Xu et al., 2010). Another study used the LC-MS/MS method with sufficient methodological validation, and simultaneously determined 10 metabolites in the plasma of dogs that had taken DCQD orally, including rhein, emodin, aloe-emodin, chrysophanol, rheochrysidin, naringin, naringenin, hesperidin, honokiol, and magnolol (Yu et al., 2009).

Table 1 summarizes the main metabolites present in DCQD.

**TABLE 1:** Main compounds in DCQ decoction.

Origin	Compounds
Rheum officinale Baill	rhein, emodin, aloe emodin, gallic acid, 2-cinnamoy-glucose, (−)-epicatechin-3-O-gallate, galloyl-neohesperidoside, physcion-8-glucoside, sennoside B glucogallin, sennoside A, 1,6-digalloy-2-cinnamoy-glucose chrysophanol-8-O-β-D- glucopyranoside, (−)-epicatechin
Magnolia officinalis	magnolignan A, magnolignan D, honokiol, magnolol, isomagnolol
Citrus × aurantium L	eriocitrin, neoeriocitrin, isonaringin, naringin, hesperidin, hesperetin-7-glucoside neohesperidin, naringenin, poncirin, hesperetin

Tang ground the DCQD particles into a fine powder and dissolved 100 mg of it in 50 mL of 65% methanol, and then used a reverse phased-C18 column (150 × 4.6 mm) high performance liquid chromatography (HPLC) method to simultaneously determine the contents of the main metabolites in DCQD. The average values of the three tests were as follows: hesperidin, 11.06 mg/g; naringin, 3.83 mg/g; emodin, 2.48 mg/g; aloe-emodin, 1.73 mg/g; honokial, 1.26 mg/g; magnolol, 1.11 mg/g; rhein, 0.86 mg/g; and chrysophanol, 0.55 mg/g (Tang W. et al., 2008). The methodology has validated the above results and is reliable and efficient.

However, the medicinal materials studied above are limited to Sichuan, China. The metabolites in Chinese medicinal materials from different origins or batches may vary. It is necessary to conduct the same study on Chinese medicinal materials from different origins and batches. Moreover, in the AP pathological model, the quantitative analysis of other contents in DCQD needs further study.

### Pharmacokinetic analysis

The pharmacokinetic characteristics of multiple metabolites in TCM are an important part of research on the material basis of traditional Chinese medicine metabolites prescriptions. Determining the pharmacokinetics of TCM is beneficial for improving the efficacy of traditional Chinese medicine and reducing toxicity and side effects.

### Absorption

The pharmacokinetic parameters of the main metabolites were determined using liquid chromatography-mass spectrometry (LC-MS) in dog plasma. The study design included oral administration of DCQD at a dose of 6 g/kg, and blood samples were collected at various time points to measure the concentrations of the metabolites. The pharmacokinetic parameters, including Cmax, Tmax, AUC, and T1/2, were calculated using non-compartmental analysis (Yu et al., 2009). The pharmacokinetic parameters are shown in Table 2. Among them, magnolol had the fastest absorption rate, reaching the maximum blood concentration 0.9 h after administration, indicating that these metabolites can quickly exert their therapeutic effects. Rheochrysidin was absorbed slowly, reaching the maximum blood concentration 11.6 h after administration. Rhein had the highest bioavailability among the 10 metabolites, with an area under the concentration-time curve (AUC) of 1,124.4 ng h·mL^-1^. In addition, different combinations may affect the absorption of DCQD metabolites. A pharmacokinetic study of DCQD after oral administration to rats in different combinations revealed that within 12 h, compared with the DCQD combination group, the group treated with dahuang alone had higher average blood drug concentrations of rhein, emodin, and chrysophanol, indicating that the compatibility of traditional Chinese medicine has an impact on the absorption of individual metabolites. The T_1/2_ and C_max_ of magnolol in the group treated with houpo alone were significantly delayed, suggesting that the compatibility of traditional Chinese medicine affects its metabolic process (Gong et al., 2012).

**TABLE 2:** Partial pharmacokinetic parameters of the active compounds of DCQD in dogs administered orally at 6 g/kg (n = 6).

Compound	T_max_ (h)	C_max_ (ng·h/mL)	AUC_0→t_ (ng·h/mL)	T_1/2_ (h)
Magnolol	0.9	14.1	53.3	3.9
Emodin	1.5	8.5	30.2	2.8
Hesperidin	1.6	152.5	710.2	3.9
Naringin	1.6	91.0	361.9	3.1
Aloe-emodin	1.7	23.7	95.3	2.7
Rhein	1.9	343.0	1,124.4	2.4
Honokiol	2.2	32.5	163.8	2.1
Chrysophanol	2.5	33.4	175.8	4.3
Naringenin	3.2	22.2	99.5	3.9
Rheochrysidin	11.6	2.1	9.3	3.8

### Distribution

Experiments have shown that the metabolites of DCQD can be absorbed into the target organs of AP. When low, medium, or high doses of DCQD were given to rats with acute pancreatitis, the main metabolites of DCQD were distributed in the pancreas, lung, and intestinal tissues. In these organs and tissues, the concentrations of metabolites increased simultaneously with increasing oral doses of DCQD. When a high dose of DCQD was given, the content of rhein was high in the lung tissue, the naringenin content was high in the intestinal tissue, and the content of rhein was high in the pancreas. These differences indicate that the specific distribution of traditional Chinese botanical drug is related to the different target tissues, possibly related to the blood barrier in different tissues (Zhao et al., 2015).

### Metabolism

After a single oral administration of DCQD to dogs, the half-life period (T_1/2_) of the 10 metabolites was less than 6 h (Yu et al., 2009), suggesting that these metabolites take effect for a short time. In clinical use, attention should be given to multiple administrations at intervals to increase blood drug concentrations. Alternatively, there is currently a lack of analysis on the metabolites in bile, urine, and feces after the oral administration of DCQD, and further research is needed.

Disease conditions may affect the absorption and metabolism of different metabolites. The pharmacokinetic parameters of the above metabolites may change in the AP model. Gong (Gong et al., 2009) gave a single oral administration of DCQD to rats in a SAP experiment and reported that, compared with those in the control group, the peak concentrations (C_max_) of rhein, rheochrysidin, chrysophanol, magnolol, hesperidin, and naringin in the SAP group were all lower, among which the times to peak drug concentrations (T_max_) of rhein, rheochrysidin, and hesperidin were significantly prolonged; the average blood concentrations of rhein, rheochrysidin, and hesperidin were all increased; the AUC of rhein, chrysophanol, magnolol, hesperidin, and naringin were significantly reduced. Except for magnolol and naringin, which did not change significantly, the half-live period of the other four metabolites was prolonged. The change trends are shown in Table 3. These results suggest that SAP can affect the absorption, metabolism, excretion, and bioavailability of DCQD, which may be related to an insufficient effective blood volume during the inflammatory response.

**TABLE 3:** Trend of pharmacokinetic parameters in rats after single dose of DCQD gavage (compared to the blank control group).

	Rhein	Rheochy	Chrysophanol	Magnolol	Hesperidin	Naringin
T1/2	↑*	↑*	↑*	-	↑	-
CL/F	↓*	-	↓*	-	-	-
AUC(0-∞)	↓*	-	↓*	↓*	↓*	↓*
MRT(0-t)	↓	-	↑	↑	↓	-
Tmax	↑*	↑*	↑*	↓	↑*	-
Cmax	↓	↓*	↓*	↓*	↓*	↓*

Note: “*” indicates p < 0.05 and “-“ indicates that the change is not significant or the data is not provided in the original text.

When DCQD is used simultaneously with the proton pump inhibitor ranitidine, the T_1/2_ and T_max_ of ranitidine are prolonged, suggesting that DCQD can affect the pharmacokinetics of modern medicines. The simultaneous use of these two drugs should be avoided in the clinical treatment of AP (Tang WF. et al., 2008).

### Network analysis

Network analysis is an emerging discipline based on systems biology theory, biological system network analysis, and the selection of specific signal nodes for multitarget drug molecule design. The multi-components and multi-target characteristics of TCM are similar to the integrity, systematicity, and comprehensiveness of network analysis. Network analysis has been widely used to elucidate the physiological activity mechanism of TCM (Li et al., 2023)- (Zhao et al., 2023).

Combined with TCMSP, TCMID4, TCM and other databases, compounds with an oral bioavailability (OB) ≥ 20 and a drug-likeness (DL) ≥ 0.15 were screened, and a total of 71 DCQD chemical compositions were screened, including 30 kinds of dahuang, 33 kinds of zhishi, and eight kinds of houpo. Through the prediction of potential targets of chemical components, 535 potential genes were identified. After further enrichment through the DAVID database and signaling pathways, and screening of AP differential genes through the DAVID database, 445 potential genes were obtained (Sun et al., 2020).

**FIGURE 2:**
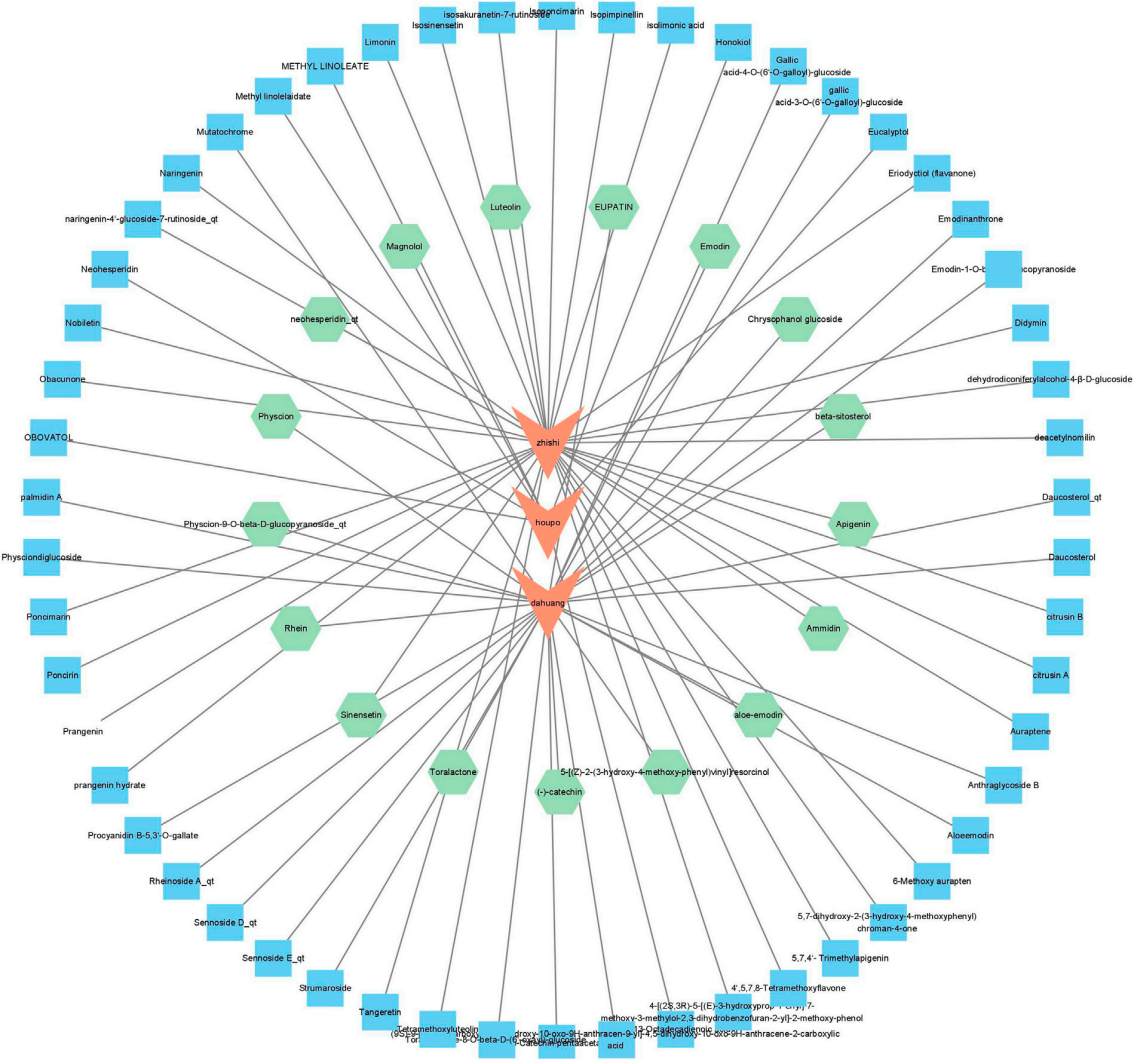
The 71 compounds in DCQD for the treatment of AP were identified based on network pharmacology prediction (the inner ring consists of 17 compounds related to the PI3K-AKT signaling pathway).

Pathway enrichment analysis revealed that the PI3K‒AKT signaling pathway was the most common pathway. Seventeen chemical components corresponding to DCQD were found via this pathway. Figure 2 shows the chemical compositions predicted by the pharmacology of the net medicine. Among them, those derived from rhubarb are (11): aloe-emodin, 5-[(Z)-2-(3-hydroxy-4-methoxy-phenyl)vinyl]resorcinol, eupantin (−)-catechin, toralactone, rhein, beta-sitosterol, emodin, physcion, physcion-9-O-beta-D-glucopyranoside_qt, and chrysophanol glucoside. Aurantii Fructus (4): neohesperidin_qt, sinensetin, luteolin, ammidin, apigenin. Magnolia officinalis (1): Magnolol (Sun et al., 2020).

In TCM theory, the main drug is the core of a prescription. The main drug used for DCQD is dahuang. Among the 17 metabolites screened, *dahuang* contains the largest number of metabolites, which aligns with the characteristics of TCM prescriptions. This finding also shows that network analysis can provide ideas for the combination of modern medicine and TCM, and to a certain extent, it shows the feasibility of TCM theory in guiding clinical medication.

It is important to note that network analysis, while providing valuable insights into the potential interactions between compounds and targets, has limitations. The predictions made by network analysis are based on computational models and databases, which may contain inaccuracies. Therefore, the results should be interpreted with caution and validated through experimental studies.

### Efficacy mechanism of some metabolites in DCQD

The basic chemical compositions of DCQD obtained by high-resolution mass spectrometry technology, combined with network analysis to predict the metabolites of DCQD in the treatment of AP, suggests that metabolites such as emodin, rhein, and luteolin in DCQD may play a therapeutic role in the treatment of acute pancreatitis. A systematic literature search was conducted across five databases (Web of Science, PubMed, Embase, Cochrane Library, and CNKI) using the refined search strategy (“emodin” OR “rhein” OR “luteolin”) AND (“acute pancreatitis”) AND (“mechanism” OR “pathway” OR “anti-inflammatory”). Studies published between 2008 and 2023 were screened, with inclusion criteria prioritizing preclinical and clinical investigations. Reviews, conference abstracts, and non-English/Chinese publications were excluded. This comprehensive search confirmed that the therapeutic effects of these metabolites on AP, particularly their anti-inflammatory and pathway-modulating activities, have been experimentally validated in prior studies.

**FIGURE 3:**
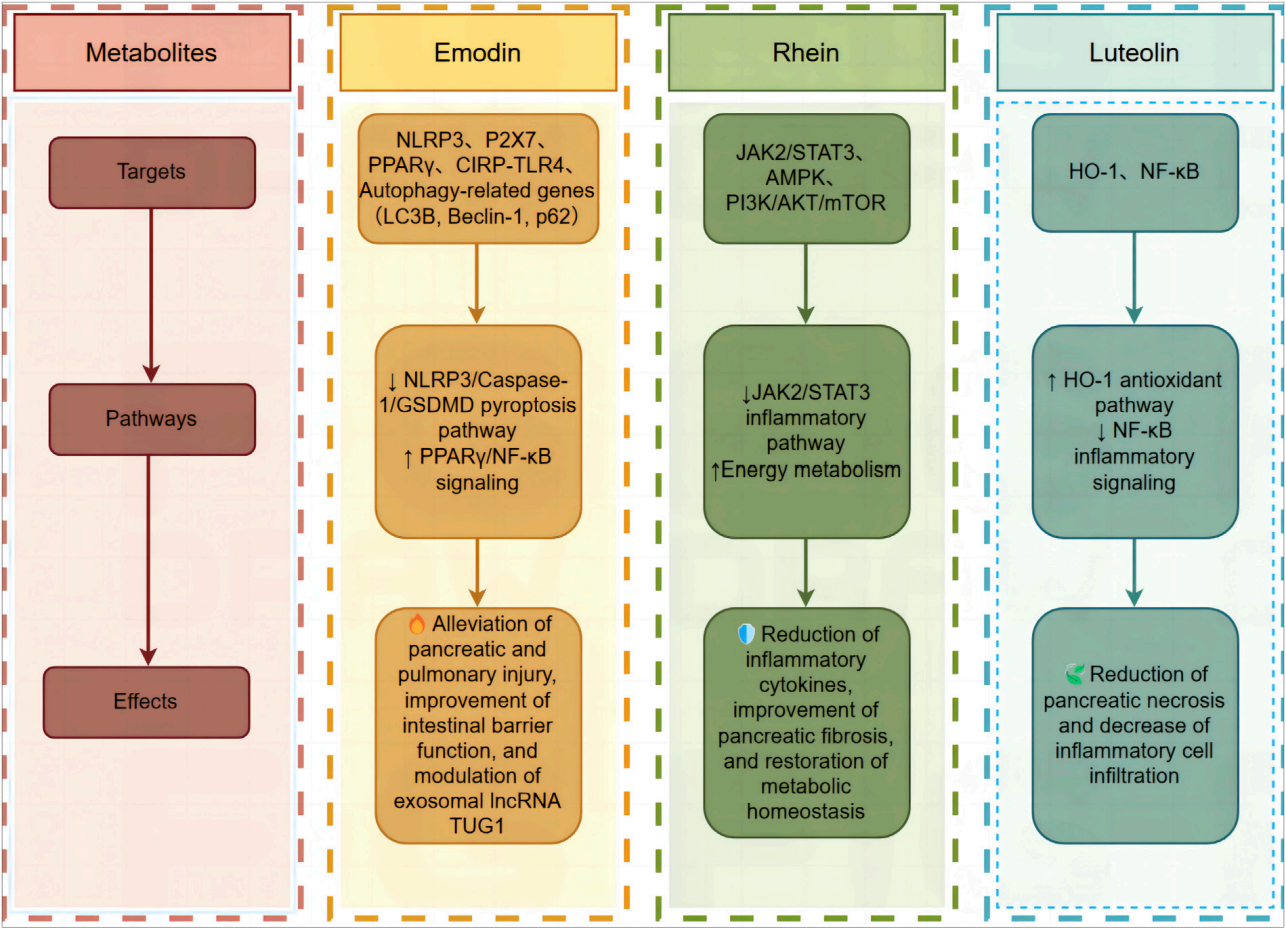
Mechanisms of key monomers in DCQD for treating acute pancreatitis.

Table 4 and Figure 3 summarizes the mechanisms of these therapeutic effects.

**TABLE 4:** The efficacy mechanisms of partial monomers in DCQD for treating AP.

Monomers used for treatment	Source	Vivo/Vitro experiments	Models	Administration method	Minimal active dose	Controls	Pharmacological effects	Mechanisms
Emodin	*Rheum officinale Baill*	Vivo	SAP rats	Gavage 10 mg/kg/d	10 mg/kg/d	Model control	Alleviated pancreas and lung injury: the histological score was reduced (p < 0.0001)	decrease the ROS activity and involved in the downregulation of NLRP3, caspase-1, and IL-18 (Xia et al., 2019)
		Vivo	AP rats	Gavage 30 mg/kg/d, 60 mg/kg/d	30 mg/kg/d	Model control	Alleviated pancreas injury: the histological score was reduced (p < 0.05). Reduced lipase and amylase levels (p < 0.01)	inhibiting the P2X7/NLRP3 signaling pathway (Zhang et al., 2019)
		Vivo	AP rats	Gavage 40 mg/kg/d	40 mg/kg/d	Model control	Alleviated pancreas injury: the histological score was reduced (p < 0.01). Reduced amylase levels (p < 0.05)and autophagic vacuole formation	Reduces transcription levels of LC3B, beclin-1, and p62 (Yu et al., 2018)
		Vitro	Rat pancreatic acinar cell lines	20 or 40 µM	20 µM	Model control	Inhibited the induction of inflammation: promoted the differentiation of Treg cells	regulation of the expression levels of cellular and exosomal lncRNA TUG1 (Wen et al., 2021)
		Vivo	AP rats	Gavage 5 mg/kg/d, 10 mg/kg/d	5 mg/kg/d	Model control	Alleviated lungs injury: the histological score was reduced (p < 0.001). Reduced levels of macrophages, MPO, and TNF-a	regulate the PPARg/NF-kB pathway (Hu et al., 2022)
Emodin	*Rheum officinale Baill*	Vivo	SAP rats	Gavage 10 mg/kg/d	10 mg/kg/d	Model control	Alleviated lungs injury: the histological score, lipase and amylase levels were reduced (p < 0.05)	inhibition of NLRP3/Caspase1/GSDMD-mediated AMs pyroptosis
		Vivo	SAP rats	Gavage 40 mg/kg/d	40 mg/kg/d	Model control, Positive control (C23)	Alleviated lungs injury: the histological score, IL-1β and amylase levels were reduced (p < 0.05), same trend as the positive group	signaling pathways (Wu et al., 2015)
		Vivo	SAP rats	Gavage 40 mg/kg/d	40 mg/kg/d	Model control	Alleviated lungs injury: reduced the histological score and neutrophil infiltration	regulate the CIRP/NLRP3/IL-1β/CXCL1 pathway (Xu et al., 2021a)
		Vivo	SAP mice	Intravenous injection 2.5 mg/kg/d	2.5 mg/kg/d	Model control	Improved pancreatic inflammation and edema, reduced paracellular permeability	Upregulates NOVEL-Rno-miR-29–3p (Yang et al., 2023)
		Vivo	SAP mice	Intraperitoneal injection 70 mg/kg/q8h	70 mg/kg	Model control	Alleviated terminal ileum injury: reduced the histological score (p < 0.001)	Promote pancreatic claudin-5 and occluding expression (Xia et al., 2012) regulated the ratio of T helper type 1 (TH1), TH2, TH17, γδ T cells, and interferon γ/interleukin 17 producing γδ T cells (Zhou et al., 2021)
Rhein	*Rheum officinale Baill*	Vivo + Vitro	SAP rats Rat pancreatic acinar AR42 J cells	Gavage 30 mg/kg/d, 7 days	30 mg/kg/d, 7days	Model control	Alleviated pancreas injury: the histological score was reduced	Inhibite JAK2/STAT3 signaling pathway (Yang et al., 2022b)
		Vivo	AP mice	Intravenous injection 4 mg/kg/d	4 mg/kg/d	Model control	Alleviated pancreas injury: the histological score was reduced	Influence riboflavin metabolism, glycerophospholipid metabolism, linoleic acid metabolism, and pentose and glucuronate interconversions pathways (Huang et al., 2020)
		Vitro	rat pancreatic acinar AR42 J cells	1 µM	1 µM	Model control	improved cell ultrastructure, especially the improvement of mitochondrial morphology	activation of PI3K/AKT/mTOR signaling pathway and activity inhibition of AMPK (Zhao et al., 2018)
Luteolin	*Citrus aurantium L*	Vivo	SAP mice	Intraperitoneal injection	100 mg/kg/d	Model control	improved inflammatory cell infiltration and necrosis in the pancreas	induce HO-1-mediated anti inflammatory and antioxidant activities, and suppress the activation of the NF-κB pathway (Xiong et al., 2017)
				100 mg/kg/d				

Emodin, a primary bioactive metabolite of DCQD, exhibits multi-target therapeutic effects in acute pancreatitis (AP) through diverse mechanisms (Dong et al., 2016)- (Zhao et al., 2018). When AP occurs, abnormal activation of pancreatic enzymes causes pancreatic neutrophil infiltration, resulting in the production of many inflammatory mediators and reactive oxygen species (ROS). Experimental studies in rodent AP models demonstrate that emodin (10 mg/kg, oral) significantly attenuates pancreatic and lung tissue damage by suppressing ROS activity and downregulating NLRP3 inflammasome components (NLRP3, caspase-1, and IL-18), thereby reducing systemic inflammation (Xia et al., 2019). This aligns with its capacity to inhibit the P2X7/NLRP3 signaling pathway, which correlates with decreased plasma IL-1β and IL-18 levels in SAP models (Zhang et al., 2019). Furthermore, emodin restores impaired autophagic flux in AP by reducing autophagic vacuole formation and modulating autophagy-related gene expression (LC3B, beclin-1, and p62) (Yu et al., 2018).

Exosomes are a type of extracellular vesicles that participate in physiological and pathological processes such as the inflammatory response, infection, and immune regulation, and affect the progression of AP (Yang Q. et al., 2022). *In vitro* experiments have shown that emodin may promote the differentiation of Treg cells and inhibit the inflammatory induction of AR42 J cells by inhibiting the expression of the exosomal lncRNA TUG1 (Wen et al., 2021).

In addition, studies have shown that emodin also has a protective effect on secondary organ damage associated with pancreatitis (Wang et al., 2009). In pancreatitis-induced lung injury, emodin reduces plasma/pancreatic exosome levels in SAP rats and inhibits the NF-kB pathway by regulating the PPARg pathway, thereby reducing the activation of alveolar macrophages and alleviating lung inflammation (Hu et al., 2022). Pyroptosis is a new form of programmed cell death that participates in inflammatory responses and exacerbates the course of the disease (Kovacs and Miao, 2017). NLRP3 inflammasomes are involved in the inflammatory process of acute lung injury, and their activation can trigger caspase-1 maturation and ultimately induce caspase-1-dependent cell pyroptosis (Wu et al., 2015). Emodin can improve AP-related lung injury and the inflammatory response by inhibiting NLRP3/Caspase-1/GSDMD-mediated alveolar macrophage (Ams) pyroptosis (Wu et al., 2022). Treatment with emodin significantly inhibited the expression of CIRP in pancreatic islets and lung tissues upregulated by SAP and attenuated SAP-activated NF-κB signaling, NLRP3 inflammasome formation, CXCL1 expression in lung-resident macrophages, and neutrophil infiltration in rat lungs. Emodin may act as an inhibitor of CIRP-TLR4 signaling, thereby improving lung function damage in SAP model rats (Xu Q. et al., 2021). Inflammatory cells can release exosome-specific inflammatory miRNAs, exacerbating dysfunction of extrapancreatic organs. Emodin may prevent lung injury in SAP rats via NOVEL-rno-miR-29a-3p expression (Yang et al., 2023).

Pancreatitis can cause intestinal mucosal barrier loss, further aggravating inflammatory and immune responses. Emodin can promote the expression of mucosal barrier-related proteins such as claudin-5 and occludin, and reduce the permeability of pancreatic paracellular cells (Xia et al., 2012). Emodin can reduce the congestion and edema of intestinal tissue and the loss of microvilli in the SAP mouse model, regulate the immune response, reduce the increase in FITC-dextran levels caused by SAP, and protect against intestinal mucosal barrier loss (Zhou et al., 2021).

Rhein has a therapeutic effect on AP. After SAP model rats were treated with rhein, the levels of inflammatory factors in the body were reduced, and the levels of proteins related to the JAK2/STAT3 signaling pathway were also decreased, suggesting that rhein may exert its anti-inflammatory effect by inhibiting the JAK2/STAT3 signaling pathway (Yang X. et al., 2022). Metabolomic analyses reveal its modulation of riboflavin, linoleic acid, and glycerophospholipid metabolism, suggesting a role in restoring metabolic homeostasis (Huang et al., 2020). Experiments have shown that rhein can improve cell ultrastructure, mitochondrial structure, and function, possibly by downregulating AMPK expression, increasing PIK/AKT/mTOR signaling pathway expression, and promoting protein synthesis, thereby improving the energy metabolism of pancreatic cells (Zhao et al., 2018). In addition, rhein has a therapeutic effect on chronic pancreatitis. In mice with experimental chronic pancreatitis, rhein attenuated the activation of pancreatic stellate cells and improved pancreatic fibrosis and pancreatic function (Tsang et al., 2013).

Luteolin has antioxidant, antiapoptotic, and anti-inflammatory properties (Sun et al., 2015). Luteolin can reduce the dry-wet ratio of the pancreas in mice with acute pancreatitis and improve pancreatic inflammatory cell infiltration and necrosis. The mechanism may involve inducing HO-1-mediated anti-inflammatory and antioxidant activity, inhibiting the activation of the NF-κB pathway, and reducing the release of inflammatory factors, thereby exerting a protective effect (Xiong et al., 2017).

Collectively, these metabolites target overlapping and distinct pathways to synergistically alleviate AP progression. However, critical gaps persist, including insufficient clinical validation of dose-response relationships, limited exploration of multi-component interactions in DCQD, and unresolved controversies regarding exosomal regulation. Future studies should prioritize translational research to bridge preclinical findings with therapeutic applications.

## Discussion and outlook

DCQD demonstrates significant therapeutic potential in AP treatment, as evidenced by its multi-component synergy, pharmacokinetic adaptability, and multi-target mechanisms. This review systematically consolidates the material basis of DCQD, emphasizing its metabolites, pharmacokinetic profiles, and efficacy mechanisms. However, several limitations necessitate cautious interpretation and further exploration.

First, while network analysis offers valuable insights into DCQD’s multi-target interactions, its computational predictions require experimental validation. For instance, databases such as TCMSP and TCMID4, though widely utilized, may contain incomplete or biased data due to inherent challenges in natural product curation. Future studies should prioritize *in vitro* and *in vivo* validation of predicted compound-target-pathway relationships to confirm their biological relevance. Second, pharmacokinetic data derived from animal models may not fully mirror human physiological responses. Variations in metabolic pathways, tissue distribution, and drug clearance between species could limit the translational applicability of these findings. Human clinical trials are essential to establish reliable pharmacokinetic parameters and optimize dosing regimens. Third, although the efficacy mechanisms of key metabolites are supported by preclinical studies, clinical evidence remains sparse. Rigorous dose-response studies and randomized controlled trials are needed to validate these mechanisms in human AP patients.

The holistic philosophy of TCM aligns with the integrative approach of network pharmacology, which bridges multi-component interactions with systemic therapeutic effects. However, the complexity of DCQD’s formulation, including variable compatibility ratios, decoction methods, and batch-to-batch heterogeneity in herbal sources, introduces challenges in standardizing its chemical profile. Advanced analytical strategies, such as LC-MS-based metabolomics combined with geographic origin studies, could enhance quality control and ensure consistency in clinical applications (Zhang et al., 2023).

Pharmacokinetics is important in terms of clinical administration routes, dosages, intervals, etc. The composition of DCQD is complex, and determining the pharmacokinetics of its main metabolites simultaneously is challenging. The pharmacokinetic parameters of different metabolites were obtained via serum mass spectrometry analysis, but further research is still needed. On the one hand, the pharmacokinetics differ between normal models and disease models. On the other hand, the pharmacokinetic parameters in the circulation cannot fully reflect the pharmacokinetic characteristics of DCQD. Therefore, it is necessary to simultaneously analyze the absorption, metabolism, and other laws of different metabolites in the AP model, and further study the pharmacokinetic characteristics of the metabolites in the intestinal cavity, intestines, and target tissues. In addition, different modes of administration, such as drug absorption after DCQD enema, are worthy of further study.

Modern research on TCM aims to use advanced analytical techniques to clarify its metabolites, establish quality control methods and standards, reveal the compatibility rules of TCM prescriptions, improve quality standards and innovate on this basis to achieve the modernization and internationalization of TCM. In recent years, with the deepening of basic research on TCM substances, some scholars have proposed the concept of integrative pharmacology in traditional Chinese medicine. Integrative pharmacology is a frontier discipline merging TCM theory with systems biology, provides a robust framework for deciphering DCQD’s holistic mechanisms. By establishing comprehensive databases of DCQD’s metabolites, elucidating their ADME profiles, and mapping their interactions with disease targets, this approach could bridge the gap between traditional knowledge and modern precision medicine. Future research should also explore dynamic pharmacokinetic-pharmacodynamic correlations to identify critical bioactive compounds and optimize therapeutic regimens (Jiashuo et al., 2022)- (Xu H. et al., 2021).

In conclusion, while DCQD represents a promising therapeutic agent for AP, its transition from bench to bedside demands interdisciplinary collaboration. Addressing the aforementioned challenges through rigorous experimentation, clinical validation, and technological innovation will not only advance our understanding of DCQD but also illuminate broader principles underlying TCM’s multi-target efficacy. Such efforts are pivotal for realizing the modernization and global acceptance of traditional herbal medicine.

## Conclusion

In summary, DCQD comprises numerous metabolites. When analyzed via modern chemical analysis techniques and network analysis, DCQD is primarily composed of metabolites such as anthraquinones, flavonoids, and lignans. Metabolites in DCQD, including emodin, rhein, and luteolin, exhibit therapeutic effects in both *in vivo* and *in vitro* models of acute pancreatitis. Pharmacokinetic analysis revealed that many metabolites of DCQD in animal models exhibit rapid absorption rates and are distributed in target organs like the pancreas and intestine. Certain metabolites, such as magnolol, emodin, hesperidin, naringin, aloe-emodin, rhein, honokiol, chrysophanol, naringenin, and rheochrysidin, have short half-lives. These findings suggest that increasing the frequency of drug administration and reducing the duration of drug administration can increase the blood drug concentration during treatment. These analyses provide valuable guidance for the clinical treatment of AP with DCQD and aid scholars in understanding the material basis of its therapeutic effects on AP.

In the future, it is essential to conduct further research on the composition analysis, pharmacokinetics, and pharmacological mechanisms of DCQD and other traditional Chinese medicine prescriptions. This will enable us to delve deeper into the principles behind the efficacy of botanical drugs in treating diseases and appreciate the allure of botanical drugs.

## Abbreviations

DCQD, Dachengqi decoction; LC-MS, Liquid chromatography-mass spectrometry; AP, Acute pancreatitis; SAP, Severe acute pancreatitis; TCM, Traditional Chinese medicine; CNKI, China National Knowledge Infrastructure; HPLC, High performance liquid chromatography; AUC, Area under the concentration-time curve; T_1/2_, Half-life period; C_max_, Peak concentrations; T_max_, Time to peak drug concentration; OB, Oral bioavailability; DL, Drug-likeness; ROS, Reactive oxygen species.
